# Perceptions of HIV/STI prevention among young adults in Sweden who travel abroad: a qualitative study with focus group and individual interviews

**DOI:** 10.1186/1471-2458-14-897

**Published:** 2014-09-01

**Authors:** Anna Qvarnström, Marie G Oscarsson

**Affiliations:** Department of Health and Caring Sciences, Linnaeus University, SE-391 82 Kalmar, Sweden

## Abstract

**Background:**

Young adults are at risk for HIV/STIs because they generally have an active sex life with multiple sexual partners; moreover, they use condoms to a lesser extent. Travelling increases sexually risky behaviour, and among both women and men, sexual contacts abroad are common. Better knowledge of how young adults experience prevention efforts prior to travelling, and what they prefer, is important when planning prevention efforts to this group. Experiences of and attitudes towards prevention efforts against HIV/STI among young adults in Sweden who have travelled abroad were investigated.

**Method:**

We conducted 12 focus-group interviews and four individual interviews with young adults (20–29 years) who had travelled abroad within the last 12 months. The interviews were recorded, transcribed verbatim, and analysed using thematic content analysis. Results were discussed from a salutogenic perspective.

**Results:**

Only a few had any experience of prevention efforts against HIV/STIs. The majority welcomed the idea of prevention efforts prior to travelling and would have welcomed more, preferably short reminders or links to reliable websites, or someone professional to discuss the issue with. Most of the young adults would use the Internet to search for information. They proposed the possibility of reaching young adults through social media, and the importance of better basic knowledge in school.

**Conclusion:**

It is difficult to reach young adults before their trips abroad. Prevention efforts on HIV/STI must therefore focus on the use of established forums. Setting the foundation for a positive attitude towards condom use is needed during school years. Even social media, where there is the possibility for dialogue, should be used as an information source.

## Background

During trips abroad, trust is established quickly in new relationships when time is limited. Casual sexual contacts during overseas travel are common in both men and women, but it varies with age, type of trip, destination and length of trip [1–7]. The relatively anonymous environment, the greater proportion of free time as well as the higher alcohol consumption compared to at home can lead to several sexual partners. Also, the probability of protecting themselves against sexually transmitted infections (STIs) decreases [8]. The recent increase in certain STIs is partly due to an active sex life, greater sexual risk taking along with greater geographic mobility [9]. Competing airlines keep flight prices low and make holiday travel and international nightlife easily accessible and common [8]. For a significant portion of those who have sex while abroad, it is something that is unplanned [2].

One in four sexually active teens contracts an STI each year [10]. In Sweden, the average age for sexual debut is 16 years [11], and the average age of women diagnosed with chlamydia is 21 years and for men 23-years-old [12]. Nowadays, it has become more common to have a greater number of sexual partners outside of established relationships [13], and condoms are used to a lesser extent. This is believed to be due to the use of oral contraceptives [14], but can also be traced to too little information about STIs [15].

In Sweden, there is currently a national action plan for HIV/STI prevention for adolescents and young adults. This action plan serves as a framework to improve, monitor and develop efforts against STIs, through the school and healthcare, among other things. The efforts are to focus on increasing self-esteem, strengthening self-awareness and enhancing the ability of young people to make informed and responsible choices regarding their own health and sexuality [16].

In 2009, the largest study to date was conducted on sexuality and health among young adults in Sweden, with over 15,000 respondents aged 15–29 years [11]. It came to light that young adults (20–29 years) reported fewer encounters with prevention efforts against HIV/STIs than adolescents (15–19 years). The majority of respondents stated that they had not searched for information on the Internet, or viewed printed information about HIV/STIs or safer sex during the past year. One reason is that young adults do not encounter preventive efforts through schools to the same extent as those who are younger [11]. Another reason is that youth clinics often have an age limit of 25 years [17], which excludes young adults.

Today there are approximately 225 youth clinics in Sweden, operated by the municipality and/or county with the objective to prevent physical and mental illness among young people. In addition to an information and knowledge intermediary role, youth clinics in Sweden also aim to provide support, advice and treatment to adolescents and young adults. This is done by outreach programmes, contraceptive counselling as well as STI testing. Approximately 85% of the visits to youth clinics are by girls [17]. Youth clinics work from a salutogenic perspective where the focus is on factors that promote health.

The term salutogenesis was coined by the American-Israeli professor and sociologist Aaron Antonovsky [18] and focuses on ‘health factors’ instead of ‘risk factors’. A sense of coherence, comprising the key concepts of comprehensibility, manageability and meaningfulness, is central to salutogenesis and came about as a complement to the otherwise often problem-focused health research. It is suggested that people who successfully implement a salutogenic approach in life not only live longer and healthier, but also perceive better mental wellbeing and a higher quality of life. This perspective is used here as a tool to understand young adults’ reasoning on prevention efforts. The focus has been on health-promoting behaviour as well as factors that make young adults protect themselves against HIV/STIs, and what kinds of information they desire. We believe that this approach can be used to understand the results from an individual as well as a structural level.

Young adults are vulnerable to STIs because they generally have an active sex life, multiple sexual partners and use condoms to a lesser extent. Travelling has been identified as a factor in itself that increases risk behaviour, associated as it is with increased number of partners and less frequent condom use. There is currently very limited knowledge of travellers’ experience and attitudes towards prevention efforts against HIV/STI before travelling abroad.

The goals of this study are to describe the experiences of and attitudes towards prevention efforts against HIV/STIs among young adults (20–29) who travel abroad, as well as investigate what kind of prevention efforts young adults want before travelling abroad.

## Methods

This study is an exploratory qualitative study using focus group as well as individual interviews. Focus group interviews are a common method when people’s experiences, opinions, beliefs, values and desires are studied. The method allows respondents to generate their own questions and to approach the subject on their own terms and with their own vocabulary [19, 20]. Inclusion criteria were: young adults aged 20–29- years-old who travelled abroad within the last year. Recruitment was done in towns and cities in southern Sweden through universities, college, sports clubs, youth clinics, travel forums on the Internet as well as through snowball sampling.

### Procedure

In most of the recruitment settings, young adults were asked to participate in the study by a staff member who mediated contact with the researcher. For youth clinics, this was a midwife; at the college, a teacher; and at sports clubs, a trainer. At universities, the researcher herself informed classes about the study, inviting those interested to make contact. Respondents recruited through snowball sampling were contacted by the researcher. Brief information about the study was also published on a travel site on the Internet, whereupon those wishing to participate could contact the researcher directly.

Everyone who participated in interviews received verbal and written information about the study’s purpose and implementation before the interview. Written consent was gathered. Respondents were assured confidentiality and informed that participation was voluntary and that they could withdraw at any time without explanation. Date and place of the interviews was determined according to the respondent’s wishes, or at the university or library. Focus group interviews were carried out in gender-specific groups. By way of compensation, respondents received cinema vouchers.

The respondents were asked to complete a short questionnaire with demographic data at the time of the interview. A semi-structured interview guide was used in the interviews, covering topics related to experiences and attitudes towards prevention efforts against HIV/STI prior to travelling abroad. Interviews lasted 20–60 minutes with one researcher participating as an observer. Having an observer take notes during the session [21], who can also assist in making sure the interview does not wander off topic [22], is a recommended approach in focus group discussions. The interviews took place between September 2012 and February 2013. There were 12 focus group interviews (n = 44) and four individual interviews. A pilot interview was conducted, but no corrections were necessary and even this group was included in the analysis. Despite information about the age limits in the inclusion criteria, two of the respondents turned out to be only 19 years old (Table 1). Since they were almost in the relevant age category and had already participated in the interview, we thought it better to include them rather than disregard the whole interview.

**Table 1:** **Data about the respondents and recruitment method**

Interview no:	1	2	3	4	5	6	7	8	9	10	11	12	13	14	15	16	Total
**Participants in each group**	3	2	1	4	3	1	5	2	1	2	1	4	5	4	7	3	**48**
**Age, Median**	19	22	20	25	20	21	21	21	22	24,5	26	24,5	21	26	23	24	
**Age, Range**	19-29	21-23	20	22-28	20-21	21	20-23	21	22	24-25	26	23-28	20-21	25-26	21-25	23-25	**19-29**
**Current pursuit:**																	
Working	1	-	-	2	-	-	-	-	1	-	-	2	4	2	-	1	**13**
Studying	-	1	1	2	3	1	5	1	-	2	1	2	-	2	7	1	**29**
Unemployed	2	1	-	-	-	-	-	1	-	-	-	-	1	-	-	1	**6**
**Area of resedence**																	
Smaller community/village	1	1	-	1	-	-	3	1	-	-	-	-	-	-	-	-	**7**
Town	2	1	1	1	1	1	1	-	-	-	-	4	5	-	-	-	**17**
City	-	-	-	2	2	-	1	1	1	2	1	-	-	4	7	3	**24**
**Recruitment method**																	
Youth clinic	2	1	1	-	-	-	-	1	-	-	-	-	-	-	-	-	**5**
University	-	-	-	-	3	-	-	-	-	-	-	-	-	-	7	-	**10**
College	-	-	-	-	-	1	5	-	-	-	-	-	-	-	-	-	**6**
Sports clubs	-	-	-	4	-	-	-	-	-	-	1	4	5	4	-	3	**21**
Snowball sampling	1	1	-	-	-	-	-	1	-	2	-	-	-	-	-	-	**5**
Internet forum	-	-	-	-	-	-	-	-	1	-	-	-	-	-	-	-	**1**

The majority of respondents had either travelled in Europe (n = 32) or Asia (n = 8) on their last trip. For most of them it had lasted 7–14 days (n = 27) or 1–6 months (n = 12). The main purpose was pleasure (n = 36) or studies (n = 13), and most common was to travel with a friend/friends (n = 24) or a partner (n = 13).

### Analysis

The interviews were transcribed verbatim by the first author and analysed using thematic content analysis [23]. The transcribed material was read through to get a picture of the whole and understand the importance of the material, after which the parts that affected the study’s purpose and the overall research questions were identified. In the process of creating preliminary categories, the following aspects were taken into consideration: the number of times the subject came up, the number of people who discussed the topic, the intensity of the conversation around the topic as well as the group’s interaction around the topic.

Together, the authors discussed the content and boundaries of the preliminary categories. The categories were processed so that they would be exclusive and have an internal consistency; furthermore, the number of categories was reduced in an ongoing creative process until only seven categories remained, where all raw data for the relevant issues could be placed. During categorisation, the authors switched from looking at the text as a whole to studying parts in order to understand it in context.

The study was approved by the Regional Ethics Committee for Human Research, Faculty of Health Sciences, Linköping University, Sweden (Dnr 2012/243-31). The authors have followed the RATS guidelines for qualitative research.

## Results

The results are presented in three main categories: Personal experience of prevention efforts prior to travelling abroad, attitudes towards prevention efforts prior to travelling abroad, as well as prevention efforts wished for by young adults.

### Personal experience of prevention efforts prior to travelling abroad

Very few of the young adults had personal experience of having encountered any prevention efforts prior to travelling abroad, and had not expected it either. Most of them had not received any oral or written information or any form of intervention.

*A: “From the trip I was on, there was absolutely nothing / … /”.*

*B: “No, I can only agree / … / nothing specific on that particular trip”.*

*C: “Nah, you never receive any info before you travel abroad”.*

*D: “I don’t think so either, I’ve travelled a few times as well, both with friends and recently now with my girlfriend and I don’t remember receiving any information”. (Interview no. 4 with four men, 25, 25, 28 and 22-years-old)*

Since many trips today are booked without any personal contact with travel agencies or other actors who would be able to provide information aimed at travellers, the young adults never expected to get information.

*“I don’t know who would provide the information because you just buy a ticket and then you go”. (Interview no. 4, Male, age 28)*

Through television commercials and brochures, many were aware of the risk of getting hepatitis abroad since the hepatitis vaccination was recommended for many destinations. However, only a few encountered any prevention efforts against other STIs.

*“The only thing I noticed was not actually to do with sexuality, it was just this, that you should get vaccinated against hepatitis. / … /”. (Interview no. 7, Male, age 20)*

Those who had encountered any efforts were essentially those who themselves actively sought information by contacting, for example, a vaccination clinic or youth clinic.

Many felt that there was no obvious place where young adults 20–29-years-old could be reached by preventive measures in a natural way. Several felt that it was problematic that they, and others in their age group, did not know where they should go if they wanted information or to get tested for STIs.

*“That’s a bit difficult too, to find places. Before, you went to the youth clinic”. (Interview no. 7, Male, 23 years)*

*“Now, it’s been quite a while since I got myself tested for anything, but that’s because I don’t know where I should go”. (Interview no. 15, Female, 25 years)*

### Attitudes towards prevention efforts prior to travelling abroad

The majority of respondents were in favour of prevention efforts against HIV/STIs before travelling abroad and wished for more. Most felt that the information was needed, and they were generally positive about outreach efforts.

*“There are always new diseases, so you always need to get new information / … / More information is positive”. (Interview no. 11, Female, 26 years)*

One man was negative towards getting information about HIV/STIs before the trip. He was not adverse to information and education about HIV/STIs but felt that it would be more natural if schools addressed this issue.

*“It should feel a bit like a parent who is trying to educate someone in some way. / … / If I were to get knowledge somewhere, then I would have liked to have had it during my sex education class at school rather than getting it in the mail from an airline company”. (Interview no. 7, Male, age 20)*

### Prevention efforts wished for by young adults

Many wished that they had encountered prevention efforts against HIV/STIs in the form of short reminders, links to where they could find information or through opportunities for discussion. They also called for better basic knowledge in schools and emphasised the importance of being involved in the learning process.

The young adults felt that the information could be beneficial if it was present where travellers sought general advice during preparation for their trips, such as in travel magazines, medical information service, on the reservation page or the travel agency. It was suggested that a link could be included with the reservation so that the traveller him/herself could read further. They wanted information about various STIs as well as about how healthcare worked in different countries. Many were positive about the opportunity to have a dialogue about the topic in order to increase their knowledge and understanding.

*“I think about social media in general, not just stand and shout out the message, but being able to discuss it. Maybe there’s a Facebook page you can find more information or to talk to someone, email or have a chat or something like that”. (Interview no. 10, Female, 25 years)*

They argued that by having information about HIV/STIs available and visible in the community, it would be like a reminder for young adults to take their own initiative to seek more information or contact healthcare services. The first step is to attract attention, which can then lead the individual further, from thought to a change in behaviour.

For personal contact, it was desirable to obtain information from a person with whom they could identify with, in an environment they felt comfortable in. The person should be authoritative, competent, serious and straightforward. A tour guide or a person who works at the resort was one example of someone who could provide such information.

*“Those who are actually there, know how it is; they’ve seen how young people are. They know what can happen, those who are there”. (Interview no. 4, Male, 22 years)*

Even conversations and knowledge from friends were emphasised. Talking from personal experience, which created a strong sense of identification, could offset lack of knowledge about the subject.

*“I think it’s easier to listen to someone your own age. Well, I don’t know, it’s both maybe. If you talk to someone who is older… you probably have a little more trust in someone who is more experienced, so to speak. But, I think it is easier to communicate with someone your own age”. (Interview no. 16, Male, 25 years)*

Many called for more frequent, comprehensive and better targeted information during school, which was perceived by many as a natural place to reach young people. Given that many young adults travel abroad after graduation, the last year of high school was viewed as an appropriate time to provide information on HIV/STIs.

*“Yes, at the end (of high school) when everyone asks, ‘what will you do afterwards?’ , you think that you want to go abroad. If you get some information then, you can take it to heart”. (Interview no. 8, Female, 21 years)*

Because individuals are receptive to information at different times, many felt that it needed to be conveyed continuously. Reminders and recurring information were important ways to get their attention and allow for the information to be absorbed. One negative aspect of the information was if it was too exhaustive and took too long to read or absorb.

*“Also, if you’ve gotten the information in school when you were in eighth grade, a lot has happened since then. Both with the information they received then and also with you. How receptive were you then?” (Interview no. 10, Female, 25 years)*

*“It should be like a tweet, that much you could handle…” (Interview no. 14, Male, 26 years)*

There was also a wish that the information would focus on the positive aspects of sexuality and benefits of protecting oneself and staying healthy, and thereby change attitudes towards condoms among young people.

*“It is always about condoms, ‘use a condom to not get sick’. Instead, you could promote it more and make ads ‘have some fun, use a condom’. Kind of make it a fun thing instead of doing it as a shield that you put on…”. (Interview no. 7, Male, age 20)*

## Discussion

Young adults had little experience of being reached by prevention efforts against HIV/STI prior to travelling abroad. The majority had a positive attitude towards prevention efforts and would have appreciated getting information about HIV/STI. They highlighted the importance of communicating interactively on this subject, preferably through school, social media and other forums where there is the possibility of dialogue. In our interpretation of the results, issues of meaningfulness, comprehensibility and manageability are central concepts for understanding the young adults’ attitudes towards prevention.

Foreign travellers are an important target for prevention efforts [4, 16], but due to the heterogeneity of the group, they are difficult to reach. In Sweden, both young adults and foreign travellers are prioritised in national efforts against HIV/STIs. It is therefore noteworthy that our results show limited experience of HIV/STI prevention, highlighting the importance of adapting prevention efforts according to the target group.

According to the national action plan that guides prevention efforts, focus should be on increasing self-esteem, strengthening self-awareness and enhancing the ability of young people to make informed and responsible decisions regarding their own health and sexuality [16]. This approach relates to the salutogenic concept of meaningfulness whereby a sense of meaningfulness is the ground on which motivation to take responsibility for oneself and invest time and energy arises [18]. Motivation to take personal responsibility is something that can be gleamed in the young adults’ discussions. We believe that is important that young adults seeking to take control of their own sexual health, for example by testing for HIV/STI, are supported in this. Although most young adults who participated in the study were in the youth clinic age group, many felt that they had ‘outgrown’ the clinics. They wanted to go somewhere else and get tested for STIs but did not know where. Such a predicament can be made easier if young adults are given information beforehand on where they should go when they no longer can or wish to visit the youth clinic [24].

The majority of respondents in our study felt that it was positive with information on HIV/STIs before travelling abroad, similar to other studies [25]. It is in the feeling of being a participant in the events that form one’s daily life and future that the motivation component exists. When stimuli are perceived as expected, ordered and cohesive, a feeling of comprehensibility arises [18]. We believe that prevention efforts among young adults should be based on facts and real risks, and raise awareness about their personal control and responsibility for their own health. As a result, the sense of control over one’s own situation is strengthened.

The young adults felt that the more information the better. They were more positive about brief information, and believed that the opportunity to discuss the subject with someone knowledgeable would increase understanding. The use of social media was highlighted during the interviews as an effective way to reach young adults, in particular because of the interactive possibilities. Furthermore, other media forms were also viewed as important forums for disseminating information and influencing attitudes [26, 27]. The young adults felt that television commercials for the hepatitis vaccine had made a great impression on them and made them ‘think’ before travelling abroad. The young adults of today have grown up with a huge influx of information and are used to taking in information through different types of media where interaction is also possible. Several participants in our study preferred this. We believe it is reasonable to adapt the information channels in response, thus investing more in interactive media where there is opportunity for dialogue.

The school is an important source and a natural place to share information with adolescents and young adults, where many wish to receive sex education [26], as emphasised by the young adults in our study. The topic has been compulsory in Swedish schools since the 1950’s [28]. However, studies [26, 29, 30] have shown that it is inadequate, and the quality and content varies between different institutions [31]. This means that many pupils leave school without adequate knowledge about HIV/STIs. Despite this problem, the young adults did not express hopelessness when talking about their experience of lack of information or discussions at school. Rather they expressed comprehensibility: they had wished for more information, but the scope of what had been taught was in line with their expectations. During the focus group interviews, many had positive expressions about being able to discuss the topic. They would have liked similar discussions in small groups during their school days.

The young adults felt that the positive aspects of condom use should be emphasised rather than marketing it as a protection against disease, which was perceived negatively. Not using condoms is not necessarily related to insufficient knowledge or deliberate carelessness about one’s health. Cook [32] discusses the conflict between social norms, femininity and the initiative to use condoms among young women. Condoms can be associated with casual sex or promiscuity, whereupon the initiative to use condoms may be perceived as offensive by the partner. It can also bring attention to one’s sexual history, which is not always desirable [32]. Similar associations have also been seen in other studies where it is described as more embarrassing to buy condoms than to actually use them [33]. This conflict can be understood through Antonovsky’s theory as a feeling of unpredictability and a lack of comprehensibility that can be difficult to relate to. Attitudes towards condoms and safe sex are an area that needs more attention, particularly in sex education in schools [31].

Some of the focus groups became smaller than planned because of difficulties to coordinate times that suited everyone. Being unable to fit four of the respondents into a group, we conducted them as individual interviews. A group size of about 4 to 8 persons is deemed appropriate in the focus group interviews to obtain meaningful interactions between the participants [19]. In our study, 1–7 people were interviewed on each occasion. This may have influenced the content of the interviews. During the focus group interviews, less intervention and follow-up questions were required by the researcher to move the conversation forward than during the individual interviews. We chose to use semi-structured interviews, which are best suited for exploratory studies where new knowledge is sought; at the same time there are a number of default themes/topics for discussion [34].

Since the study has a qualitative design, it is not generalisable. However, our results can illustrate what young foreign travellers encounter as far as prevention efforts against HIV/STIs, how they perceive it and what kinds of information they wish for. The results can serve as a complement to other studies on experiences and attitudes towards prevention efforts and are applicable to similar contexts.

## Conclusions

It is difficult to reach young adults with prevention efforts against HIV/STIs before travelling abroad. Therefore, established forums must be used to reach this group. We believe that the use of social media for information dissemination, where there is also the opportunity for interaction could be utilised more, which was something the young adults requested. Providing sufficient basic knowledge in school is also important. The young adults wished that sex education in schools focused more on discussions and dialogue. Suitable educational tools to aid this process are desirable.

The school and the media have an important role in changing attitudes towards condom use and safe sex. Hence, there is a need for discussions, campaigns and information that emphasises the positive aspects of condoms, and what barriers there may be for usage. Having discussions based on changing attitudes and behaviour, both on the individual and the societal level, are also important.

Clearer strategies on how the transition from youth clinic to other clinics should take place are needed. This is in order to avoid a situation where young adults do not know where to go to get tested or get advice about their sexual health prior to travelling abroad as well as on other occasions.

We suggest that a salutogenic perspective focusing on resources, health and preventative behaviour is important when planning prevention efforts to young adults. People with high levels of comprehensibility, manageability and meaningfulness in their lives stay healthier despite stressful situations. The young adults in our study clearly expressed a desire for participation and active involvement. We suggest that future studies, as a complement to focusing on young adults, even including those nearest, such as partners and immediate families.
